# Identification of shared biomarkers and potential therapeutic targets for antiphospholipid syndrome and recurrent miscarriage by integrated bioinformatics analysis and machine learning

**DOI:** 10.3389/fmed.2025.1639277

**Published:** 2025-09-23

**Authors:** Su Zhang, Yifang Zhang, Jing Xu, Weitao Hu, Xiaolan Huang, Xiaoqing Chen

**Affiliations:** ^1^Department of Rheumatology, The Second Affiliated Hospital of Fujian Medical University, Quanzhou, China; ^2^Department of Gastroenterology, The Second Affiliated Hospital of Fujian Medical University, Quanzhou, China; ^3^Department of Gynecology and Obstetrics, The Second Affiliated Hospital of Fujian Medical University, Quanzhou, China; ^4^Department of Reproductive Medicine, The Second Affiliated Hospital of Fujian Medical University, Quanzhou, China

**Keywords:** recurrent miscarriage, antiphospholipid syndrome, bioinformatics analysis, immune infiltration, machine learning, biomarker

## Abstract

**Background:**

Antiphospholipid syndrome (APS) is a group of clinical syndromes of thrombosis or adverse pregnancy outcomes caused by antiphospholipid antibodies that can increase the probability of miscarriage occurring in pregnant women. However, the mechanism of recurrent miscarriage (RM) induced by APS is not fully understood. The aim of this study was searching for potential shared genes in RM and APS.

**Methods:**

We downloaded the APS and RM datasets from the GEO database and conducted differential expression analysis to obtain differentially expressed genes (DEGs). Their common DEGs were then identified. Functional enrichment analyses were performed on the common DEGs, follow by the construction of protein–protein interaction (PPI) networks. Next, machine learning was utilized to screen for their common key genes. Receiver operating characteristic curves (ROC) were applied to assess the diagnostic value of key genes. In addition, we performed immune infiltration analysis to understand the changes in their immune microenvironment. Subsequently, the Drug Gene Interaction Database (DGIdb) was searched for potential therapeutic drugs. Finally, the expression of key genes was verified by clinical samples.

**Results:**

We identified a total of 52 common DEGs. Functional enrichment analyses indicated that neutrophil extracellular trap formation, cellular and molecular imbalances in the immune system may be a common mechanism in the pathophysiology of APS and RM. Machine learning identified *CCR1*, *MNDA*, *S100A8* and *CXCR2* as common key genes. The key genes were highly expressed in both APS and RM. In addition, we utilized the Drug Gene Interaction Database (DGIdb) to screen for potential therapeutic drugs targeting the key genes. Finally, we validated the expression of key genes by immunohistochemical staining and RT-qPCR.

**Conclusion:**

*CCR1*, *MNDA*, *S100A8* and *CXCR2* are shared biomarkers between RM and APS. Meanwhile, our study further elucidated the biological mechanism between APS and RM.

## 1 Introduction

Miscarriage is a frequent complication of pregnancy with an incidence of approximately 15–25% and is defined as the spontaneous termination of pregnancy between the start of conception and the 24th week of gestation (1, 2). The European Society of Human Reproduction and Embryology (ESHRE) guidelines define recurrent miscarriage (RM) as two or more consecutive clinically recognized pregnancy failures (3). RM affects about 2–4% of couples (4, 5). Tragically, the greater the number of miscarriages, the greater the chance of RM occurring (6). To date, a number of causes of RM have been revealed, ranging from gestational age, hormonal and metabolic disorders, anatomical factors, immunologic factors, hereditary/secondary thrombosis and other factors (1). Nevertheless, these risk factors are still undetected in nearly 50% of miscarried pregnancies (7, 8). RM has become a major issue in the field of assisted reproduction due to its complex etiology that seriously affects the physical and mental health of patients and their couples.

Antiphospholipid syndrome (APS) is a severe systemic autoimmune disease characterized by abnormal positivity for antiphospholipid antibodies (aPL), including lupus anticoagulant (LA), anticardiolipin antibodies (aCL), and anti-β2-glycoprotein 1 antibodies (aβ2-GP1). Clinical manifestations of APS include vascular thrombosis, obstetrical complications, and thrombocytopenia (9). APS can result in microvascular thrombosis of the chorionic plate at the maternal-fetal interface, leading to RM and even stillbirth (10). APS is currently the most common treatable cause of RM and is diagnosed in 15–20% of RM patients (11, 12). The underlying connection between APS, thrombosis, and RM lies in the prothrombotic state induced by aPL. These antibodies promote thrombosis by activating endothelial cells, platelets, and monocytes, and by disrupting the natural anticoagulant systems. This hypercoagulable state can cause placental insufficiency, infarction, and inadequate blood flow to the developing fetus, ultimately resulting in pregnancy loss (13, 14). Miscarriage rates can range from 24 to 60% if aPL-positive patients do not receive appropriate treatment or intervention (12). These findings imply that infertile patients with APS are at significant risk for RM due to both thrombotic and inflammatory pathways.

In addition to microvascular thrombosis, RM in APS patients involves multiple mechanisms. A variety of pathways trigger the activation of T cells in APS patients, as well as the production of cytokines, thus affecting the normal regulation of the immune system and disrupting the immune balance (15). In addition, aPL can inhibit the migration or invasion of chorionic villus cells, reduce the expression level of complement regulatory proteins, activate complement on the surface of trophoblast cells, and trigger inflammatory responses (16). Furthermore, the formation of microvascular thrombi can severely affect endometrial peanut and early embryo implantation, ultimately leading to implantation failure, infertility and spontaneous abortion in women (17, 18). These findings strongly support the link between APS and RM. However, the underlying mechanisms remain poorly understood, necessitating the study of their common pathophysiologic mechanisms.

In this study, we identified key genes shared by RM and APS by screening with bioinformatics and machine learning methods. We also evaluated the diagnostic value of these key genes and characterized potential therapeutic agents. It is expected that this work will provide new insights and directions for understanding the association between these two diseases.

## 2 Methods and materials

### 2.1 Data acquisition and pre-processing

The GSE102215 (19), GSE50395 (20), GSE22490 (21) and GSE165004 (22) microarray datasets were obtained from the GEO database.^1^ GSE102215 and GSE50395 are based on the GPL16791 and GPL4133 platforms, respectively. While GSE22490 and GSE165004 are based on GPL570 and GPL16699 platforms. After excluding male samples in GSE102215, peripheral blood samples from 6 antiphospholipid syndrome (APS) patients and 6 healthy control (HC) individual were finally included. GSE50395 contained peripheral blood samples from 3 APS patients and 3 HC individual. GSE22490 consisted of placenta samples from 4 recurrent miscarriages (RM) and 6 elective terminations (HC). And GSE165004 contained endometrial tissue samples from 24 RM and 24 HC. Among them, GSE102215 and GSE22490 served as the training cohorts while the other two were used as the validation cohorts. The details of the datasets included in this study are presented in Table 1.

**Table 1:** The details of the datasets included in this study.

Dataset	Platform	Species	Sources	Number of cases and controls	Type of cohortts
GSE102215	GPL16791	*Homo sapiens*	peripheral blood	6 APS/6 HC	Training
GSE50395	GPL4133		peripheral blood	3 APS/3 HC	Validating
GSE22490	GPL570		Placenta	4 RM/6 HC	Training
GSE165004	GPL16699		Endometrium	24 RM/24 HC	Validating

APS, antiphospholipid syndrome; RM, recurrent miscarriage.

### 2.2 Acquisition of DEGs

The GSE102215 and GSE22490 datasets were normalized and screened for DEGs by the “limma” package (23). The selection criteria for DEGs were ┃log2FoldChange┃ > 0.585 and adj-*p* < 0.05.

### 2.3 Identification of key gene modules and common DEGs

Weighted gene co-expression network (WGCNA) for GSE102215 was constructed using the “WGCNA” package (24). The scale-free co-expression network was first constructed using the “pickSoftThreshold” function to ascertain the optimal soft threshold. Subsequently, the neighbor-joining and TOM matrices were computed for hierarchical clustering analysis. The co-expressed gene modules consisted of at least 30 genes, while nonsignificant genes were assigned to grey module. Eventually, Pearson correlation analysis was performed between individual gene modules and the phenotype of APS, as well as gene significance (GS) and module membership (MM) values were utilized to identify the key gene modules associated with APS. We then defined key gene modules, APS-DEGs and RM-DEGs crossover genes as common genes (CGs).

### 2.4 Functional enrichment analysis of CGs and construction of protein interaction networks

The CGs were uploaded to the metascape online website^2^ for Gene Ontology (GO) and Kyoto Encyclopedia of Genes and Genomes (KEGG) enrichment analysis to reveal their biological functions. The STRING database^3^ and Cytoscape (version 3.9.1) were applied to construct and visualize protein–protein interaction (PPI) networks. The most important PPI networks were acquired with Cytoscape’s MCODE plugin. The genes comprising the most important networks are recognized as candidate key genes.

### 2.5 Identification of shared key genes for RM and APS

In order to filter out the shared key genes for RM and APS, we analyzed the candidate key genes using the least absolute shrinkage and selection operator (LASSO) regression. LASSO regression was performed using the “glmnet” package (25). We then tested the diagnostic value of key genes for RM and APS by receiver operating characteristic (ROC) curves. A larger area under the curve (AUC) indicates greater accuracy as a diagnostic marker.

### 2.6 External cohort validation of key genes

In order to test the accuracy of machine learning, we validated the expression of key genes in external datasets GSE50395 and GSE165004.

### 2.7 Interaction of key genes with diseases

The Comparative Toxicogenomics Database (CTD)^4^ (26) integrates data from a diverse number of gene and disease interactions. In order to investigate the relationships between key genes and diseases, we analyzed the inference scores and reference counts for key genes and associated diseases with CTD.

### 2.8 Functional analysis of key genes

We also performed GO functional annotation of key genes to understand the biological processes in which these genes work together.

### 2.9 Immune infiltration analysis

As we have previously described, immune dysregulation is an important factor in RM and APS. Therefore, we checked and compared 22 immune cell infiltrations between the APS, RM groups and the corresponding HC group via the Cibersortx online site^5^ (27). Pearson correlation analysis to reveal correlations between key genes and immune infiltrating cells.

### 2.10 Prediction of candidate drugs

Drugs interacting with key genes were gained from the Drug Gene Interaction Database (DGIdb)^6^ (28) for prediction of potential drugs for the treatment of APS and RM. The 3D structures of the obtained drugs were provided by the PubChem website^7^ (29). Interactions between candidate drugs, key genes and immune cells are demonstrated by Sankey diagrams.

### 2.11 Collection of clinical samples

We gathered paraffin sections of placental tissue from 5 RM patients and 6 patients with elective termination of pregnancy (Control group) at the Second Affiliated Hospital of Fujian Medical University. Meanwhile, we also collected peripheral blood samples from 10 APS patients and 12 peripheral blood samples from healthy individual who excluded hepatitis B, tuberculosis, diabetes mellitus, tumors, and other types of autoimmune diseases (such as rheumatoid arthritis and primary Sjögren’s syndrome).

### 2.12 Immunohistochemical (IHC) staining

We first removed the paraffin using xylene, then hydrated it through graded alcohol (100, 95, 70%), and finally washed the sections with distilled water. Next, we placed the sections in citrate buffer for antigen repair. To prevent background interference, tissue sections are treated with 3% hydrogen peroxide to block endogenous peroxidase activity, which could produce false signals. Subsequently, we added the appropriate amounts of primary antibodies (CCR1, 1:50, DF2710, affinity, China; CXCR2, 1:200, 20,634-1-AP, proteintech, China; MNDA, 1:200, 13,576-1-AP, proteintech, China; S100A8, 1:500, ab92331, abcam, UK) dropwise to the sections and placed them in a 4 °C refrigerator overnight. The next day, the sections were washed with PBS buffer and incubated dropwise with HRP-conjugated goat anti-rabbit IgG(H + L;1:10000, SA00001-2, proteintech, China) for 1 h at room temperature. The DAB working solution was then configured for the color development reaction, followed by dehydration via gradient alcohol and sealing of the sections. Finally, the sections were observed under a microscope and images were captured.

### 2.13 RT-qPCR to validate key genes expression

The specific process can be referred to our previous study (30). The primers utilized in this study are illustrated in Table 2.

**Table 2:** The primers utilized in this study.

Gene names	Primers sequences (5′ → 3′)
B-actin-F	CATGTACGTTGCTATCCAGGC
B-actin-R	CTCCTTAATGTCACGCACGAT
CCR1-F	GACTATGACACGACCACAGAGT
CCR1-R	CCAACCAGGCCAATGACAAATA
CXCR2-F	TCCGTCACTGATGTCTACCTGC
CXCR2-R	TCCTTCAGGAGTGAGACCACCT
MNDA-F	AACTGACATCGGAAGCAAGAG
MNDA-R	CCTGATTCGGAGTAAACGAAGTG
S100A8-F	ATGCCGTCTACAGGGATGAC
S100A8-R	ACTGAGGACACTCGGTCTCTA

### 2.14 Statistical analysis

All statistical analyses were carried out in R (version 4.4.3) software. The default two-tailed *p* < 0.05 is statistically significant unless otherwise noted. The flow chart of this study is shown in Supplementary Figure S1.

## 3 Results

### 3.1 Identification of APS-DEGs and RM-DEGs

**Figure 1:**
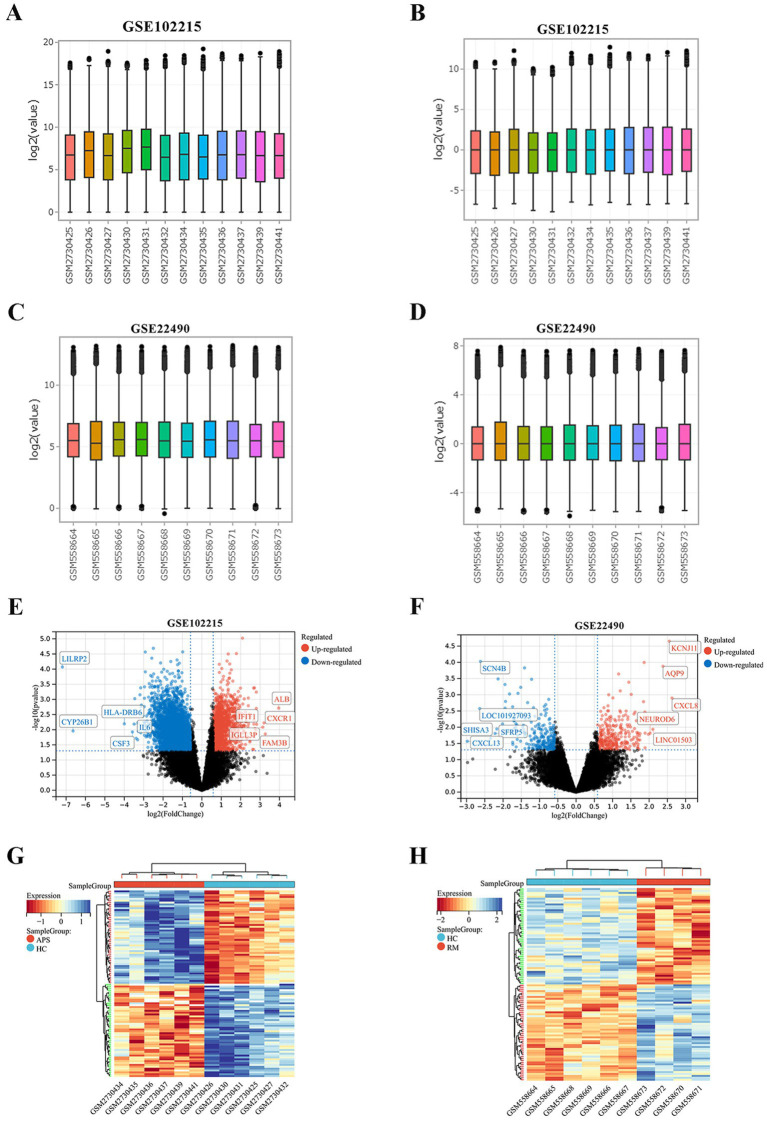
Normalization of the training cohorts and identification of DEGs. **(A,B)** Gene expression levels before GSE102215 normalization. **(C,D)** Gene expression levels before GSE22490 normalization. **(E)** The volcano plot shows the significantly DEGs of APS. Red circles represent up-regulated genes, while blue circles represent down-regulated genes. **(F)** The volcano plot displays the significantly DEGs of RM. Red circles represent up-regulated genes, while blue circles represent down-regulated genes. **(G)** The heatmap presented the expression of the top 50 DEGs in APS and HC groups. **(H)** The heatmap presented the expression of the top 50 DEGs in RM and HC groups.

We noticed that the expression values of individual samples in the APS and RM datasets before normalization varied greatly (Figures 1A,C), while the median expression of individual samples after normalization was at the same level (Figures 1B,D). This indicates that the batch effect is better removed, which is favorable for subsequent studies. Differential analysis yielded 4,492 APS-DEGs containing 2,187 down-regulated genes and 2,305 up-regulated genes (Figure 1E). A total of 502 RM-DEGs were identified, including 222 down-regulated genes and 280 up-regulated genes (Figure 1F). Figures 1G,H present the differential expression heatmaps of the top 50 up-regulated and down-regulated genes, separately.

### 3.2 The result of WGCNA and identification of CGs

**Figure 2:**
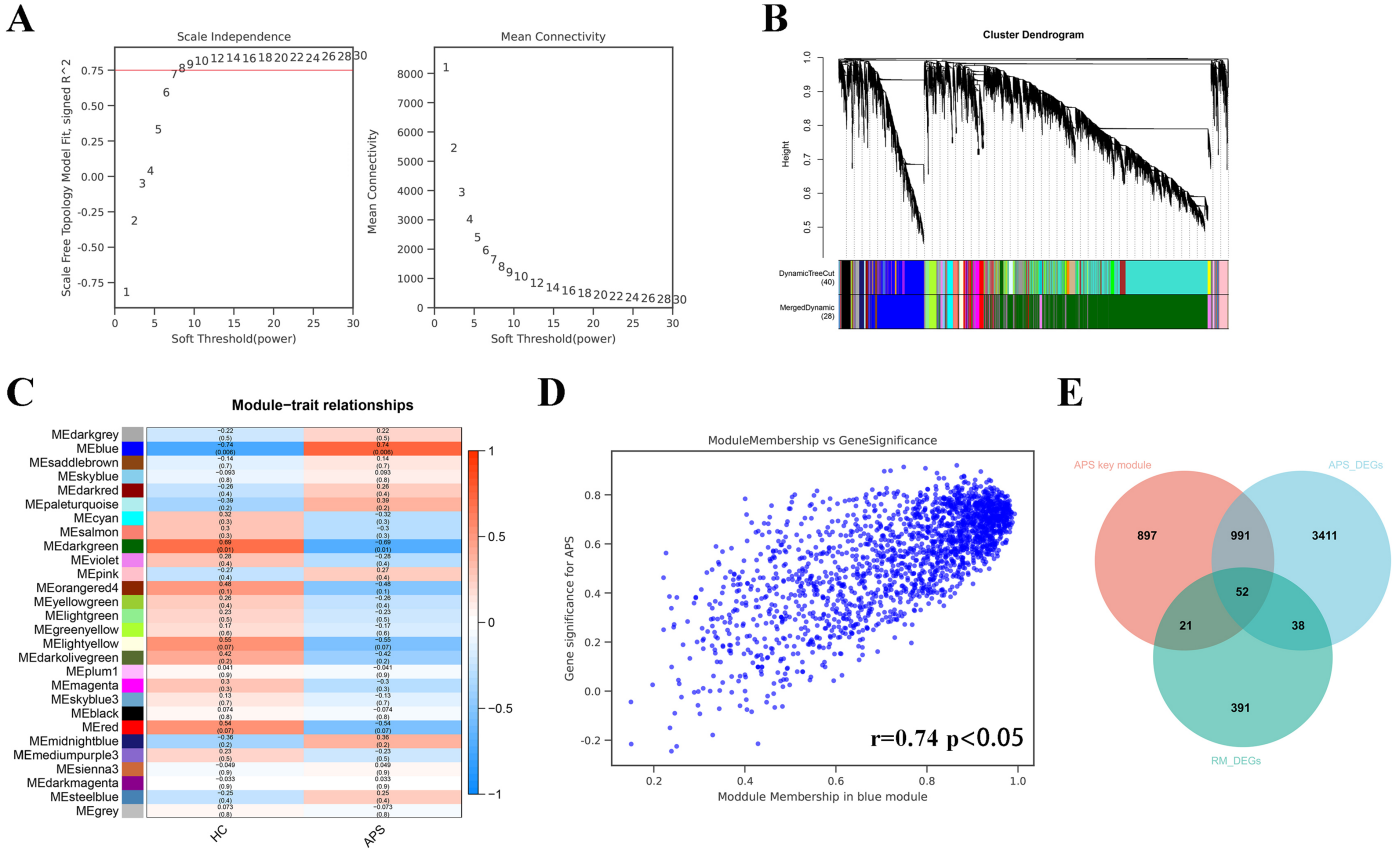
The results of WGCNA and identification of common genes. **(A)** Selection of the optimal soft threshold. **(B)** Gene module clustering tree. **(C)** Pearson test for each gene module with APS phenotype. **(D)** Correlation between blue module gene members and gene significance. **(E)** The Venn diagram presents the intersection of APS-DEGs, key module genes, and RM-DEGs. The 52 genes in the crossover section are common DEGs of APS and RM.

When the soft threshold is 8, the mean connectivity is good (Figure 2A). A total of 28 co-expression modules were derived by merging modules with distances less than 0.25. The dendrogram of module gene clustering is displayed in Figure 2B. Pearson correlation analysis between 28 gene modules and phenotypes illustrated that the blue gene module had the strongest correlation with APS (r = 0.74, *p* < 0.05; Figures 2C,D). Therefore, we defined the blue gene module as the key gene module. By means of a Venn diagram, a total of 52 CGs were identified from the blue gene module, APS-DEGs and RM-DEGs (Figure 2E).

### 3.3 Functional enrichment analysis and PPI networks construction

**Figure 3:**
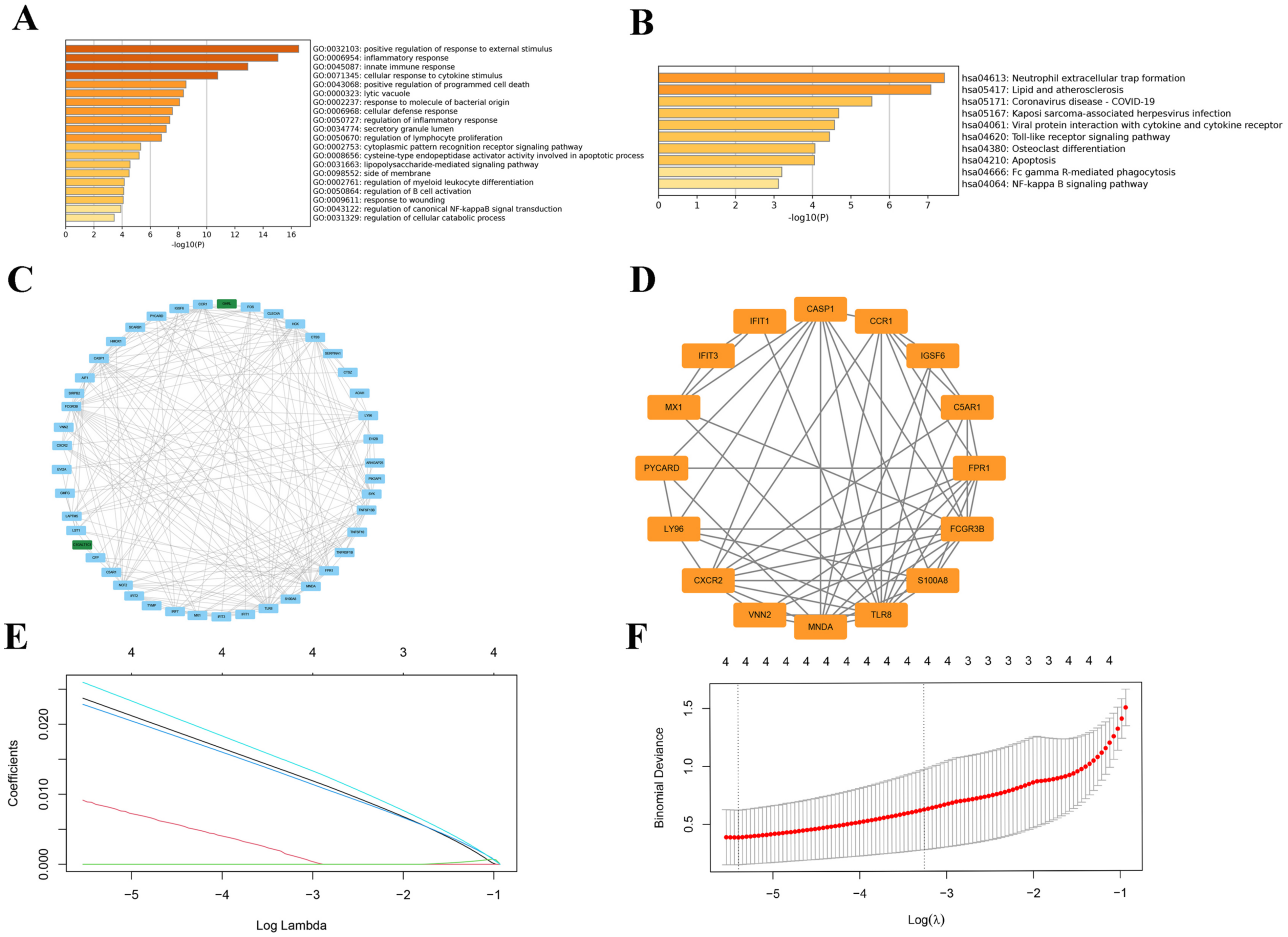
Enrichment analysis and PPI networks of common genes and identification of key genes. **(A)** GO enrichment analysis of common genes. **(B)** KEGG enrichment analysis of common genes. **(C)** PPI networks of common genes. Light blue color indicates up-regulated genes and green color represents down-regulated genes. **(D)** The most important module in the PPI networks. **(E)** The LASSO regression screened four key genes. **(F)** Penalty score plot of the LASSO model, where the error line represents the standard error.

GO analysis of CGs revealed major enrichment in positive regulation of response to external stimulus, inflammatory response, innate immune response, cellular response to cytokine stimulus and positive regulation of programmed cell death (Figure 3A). KEGG analysis of CGs was most pronounced in neutrophil extracellular trap formation, lipid and atherosclerosis enrichment (Figure 3B). The PPI network constructed by the 52 CGs has 45 points and 221 edges (Figure 3C). Subsequently, we successfully validated the expression of the key genes in external datasets. The most significant PPI network got by MCODE plugin has 16 points and 56 edges (Figure 3D). These 16 points are referred to as candidate shared key genes for RM and APS.

### 3.4 Identification of shared key genes in RM with APS

**Figure 4:**
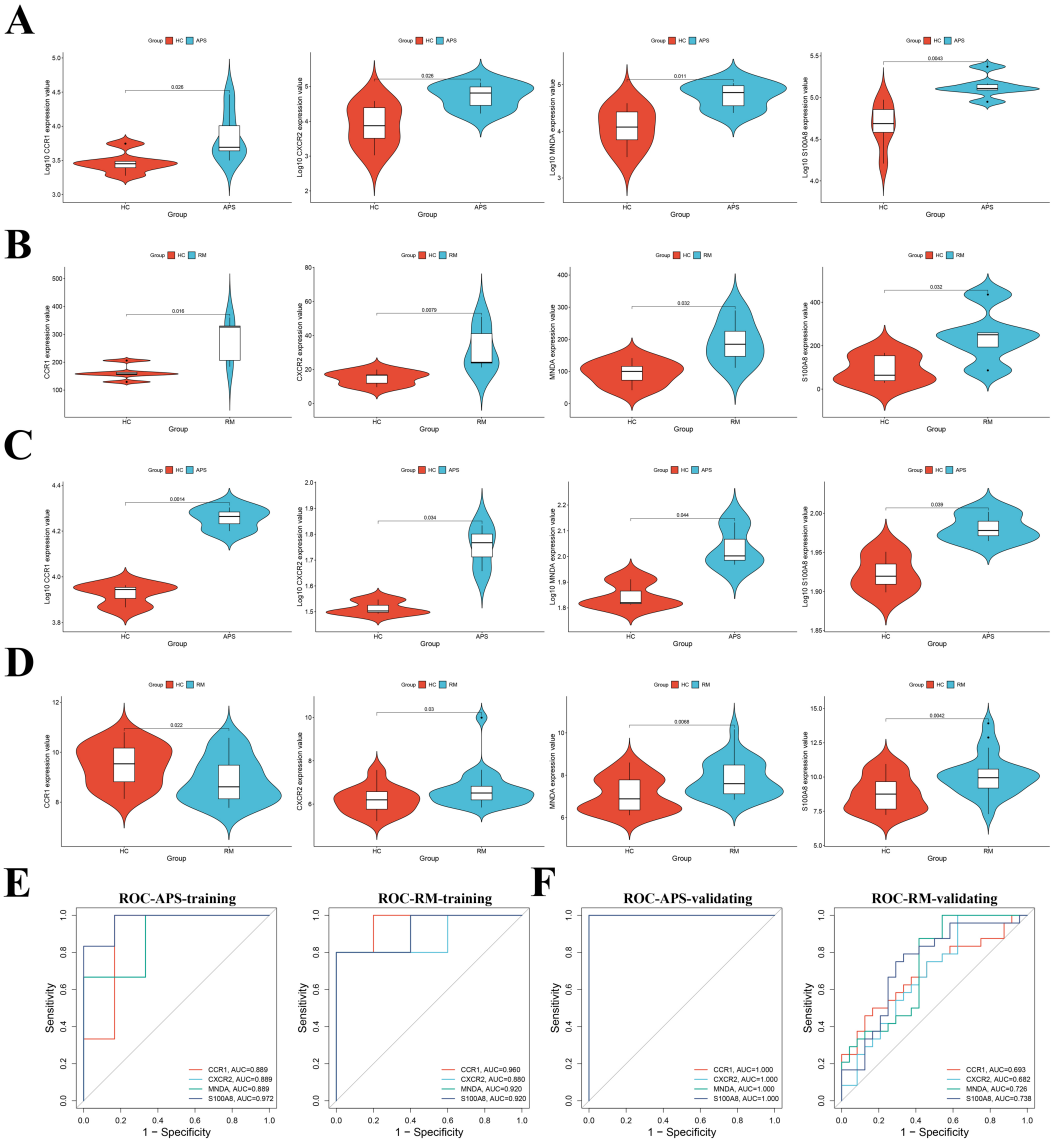
Validation and ROC analysis of key genes. **(A)** Differential expression of key genes in the APS training set. **(B)** Differential expression of key genes in the RM training set. **(C)** Differential expression of key genes in the APS validating set. **(D)** Differential expression of key genes in the RM validating set. **(E)** ROC analysis of key genes in APS and RM training sets. **(F)** ROC analysis of key genes in APS and RM validating sets.

The LASSO regression analysis of the 16 candidate key genes resulted in four genes, namely *CCR1, MNDA, S100A8* and *CXCR2* (Figures 3E,F). These four genes are the shared key genes in RM and APS. Table 3 exhibits their descriptions and functions. Subsequently, we observed that the expression of all key genes was upregulated in both the training and validation cohorts of APS and RM (Figures 4A–D). Meanwhile, ROC analysis displayed that all key genes had AUC values greater than 0.5 in both the training and validation sets, which indicates that the key genes have a favorable value in the early diagnosis of APS and RM (Figures 4E,F).

**Table 3:** The key genes and their functions.

Gene symbol	Description	Function
*CCR1*	C-C chemokine receptor type 1	It is a receptor in the immune system that plays a role in the regulation of immune responses and inflammation by binding to specific chemokines, which are signaling molecules involved in the recruitment of immune cells to sites of infection or injury. CCR1 is involved in various immune processes, including the migration of monocytes and other leukocytes (39, 42, 44).
*CXCR2*	C-X-C chemokine receptor type 2	It is a receptor in the immune system that primarily binds to C-X-C chemokines, particularly IL-8 (interleukin-8), and plays a key role in the recruitment of neutrophils and other immune cells to sites of inflammation or infection. CXCR2 is involved in various immune and inflammatory processes, including the regulation of neutrophil trafficking and the body’s response to infection and injury (65, 83).
*MNDA*	Myeloid Nuclear Differentiation Antigen	It is a protein expressed in myeloid cells, such as neutrophils and monocytes, and is involved in immune responses. MNDA plays a role in the regulation of transcriptional activity and immune cell differentiation, particularly in the context of myeloid cell function. It is considered a marker of myeloid cell differentiation and is often studied in the context of myeloid leukemias and other hematological disorders (84).
*S100A8*	S100 calcium-binding protein A8	It is a member of the S100 protein family, which is characterized by its ability to bind calcium ions. S100A8 is primarily expressed in myeloid cells, such as neutrophils and monocytes, and plays an important role in inflammation and immune responses. It is involved in the regulation of various cellular processes, including cell motility, adhesion, and the inflammatory response. S100A8, along with its dimeric partner S100A9, is also referred to as calprotectin, and it has been implicated in various inflammatory diseases and conditions (52, 55, 58).

### 3.5 Interaction of all key genes with diseases

**Figure 5:**
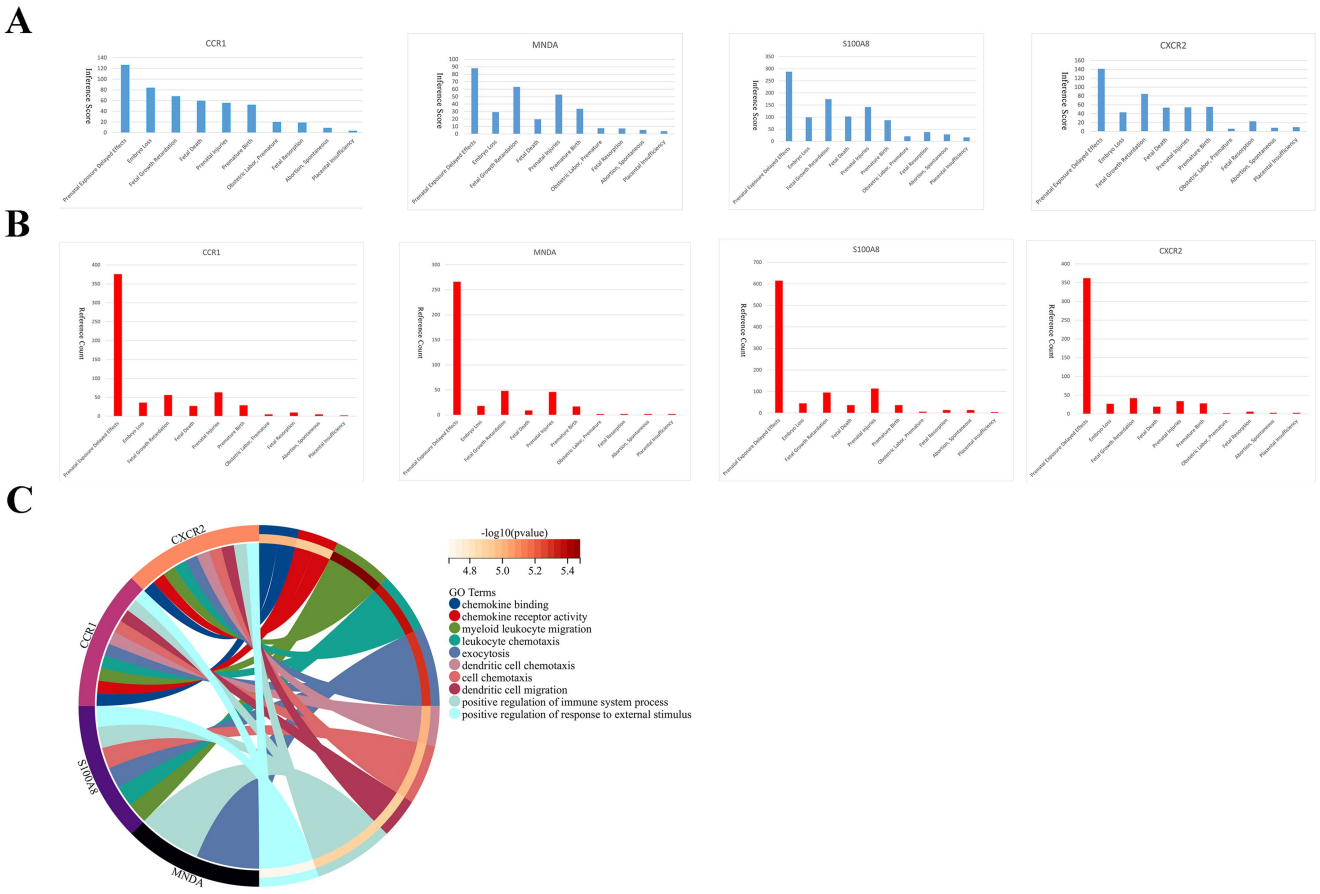
Correlation of key genes with pregnancy-related diseases and enrichment analysis of key genes. Inference scores **(A)** and reference counts **(B)** between key genes and prenatal exposure delayed effects, embryo loss, fetal growth retardation, fetal death, prenatal injuries, premature birth, obstetric labor premature, fetal resorption, abortion spontaneous, and placental insufficiency in CTD database. **(C)** Functional enrichment analysis of key genes.

We searched the CTD database and observed that key genes were related to adverse pregnancy outcomes in women (Figures 5A,B). At the same time, key genes are engaged in a variety of immune response processes (Figure 5C). These imply that key genes are participated in APS-associated RM by regulating immune processes.

### 3.6 Immune infiltration analysis

**Figure 6:**
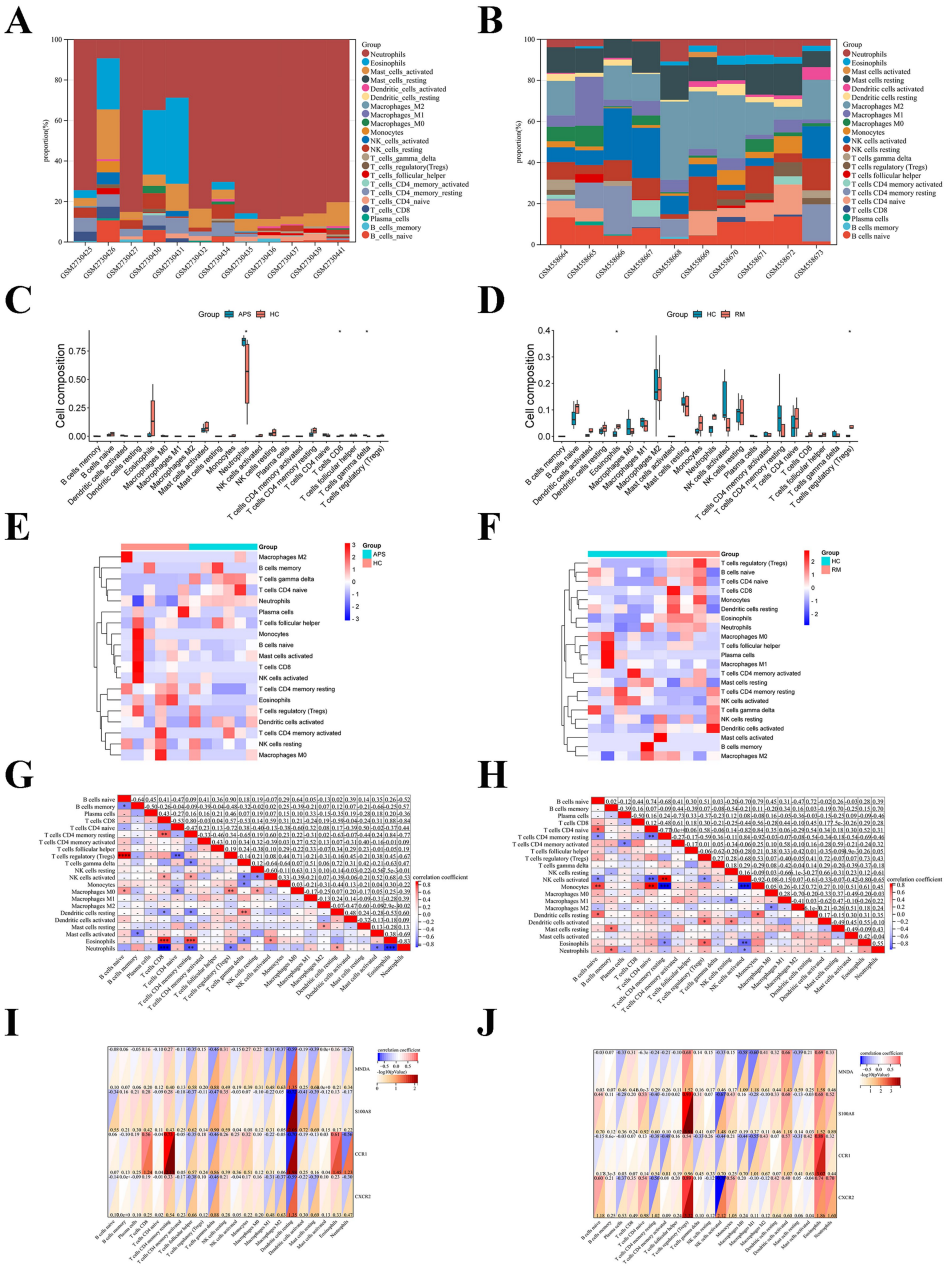
Immune infiltration analysis of APS and RM. The stacked diagrams display the immune infiltration of each sample in APS **(A)** and RM **(B)**. The box plots illustrate comparison of immune cells in APS **(C)** and RM **(D)**. Heatmaps show the distribution of immune cells in APS **(E)** and RM **(F)**. Heatmaps presented correlation between immune cells in APS **(G)** and RM **(H)**. Correlation between immune cells and key genes in APS **(I)** and RM **(J)**. Noted: * represents *p* < 0.05.

As mentioned earlier, immune dysregulation may be an essential cause of recurrent miscarriages in APS patients. Immune infiltration analysis indicated that neutrophils were the major immune infiltrating cells in APS (Figure 6A), and M2 macrophages and NK cells were the major immune infiltrating cells in RM (Figure 6B). The expression of neutrophils (*p* < 0.05) and T cells gamma delta (*p* < 0.05) were higher in APS than in HC groups; while the expression of CD8^+^ T cells (*p* < 0.05) was lower than in HC groups (Figure 6C). The expression of eosinophils (*p* < 0.05) and T cells regulatory (Tregs;*p* < 0.05) were significant higher in RM than in HC groups (Figure 6D). Figures 6E,F display in detail the distribution of various immune infiltrating cells of APS and RM. In addition, positive correlations were detected in APS between Tregs and naive B cells (*R* = 0.90, *p* < 0.0001), and CD8^+^ T cells and eosinophils (*R* = 0.88, *p* < 0.001); whereas negative correlations were observed between eosinophils and neutrophils (*R* = −0.83, *p* < 0.001), and CD8^+^ T cells and neutrophils (*R* = −0.84, *p* < 0.001; Figure 6G). Positive correlations were found in RM between monocytes and naive CD4^+^ T cells (*R* = 0.84, *p* < 0.01), activated NK cells and resting CD4^+^ memory T cells (*R* = 0.84, *p* < 0.01); whereas negative correlations were detected between monocytes and resting CD4^+^ memory T cells (*R* = −0.92, *p* < 0.001) and monocytes and activated NK cells (*R* = −0.92, *p* < 0.001; Figure 6H). Pearson test revealed a positive correlation between *CCR1* and resting CD4^+^ T cells in APS (*R* = 0.73), while *CCR1* and *S100A8* were negatively correlated with resting dendritic cells (*R* = −0.70, *R* = −0.72; Figure 6I). Furthermore, there was a positive correlation between S100A8 and Tregs (*R* = 0.93), *CXCR2* and Tregs (*R* = 0.89), and CCR1 and eosinophils (*R* = 0.88) in the RM (Figure 6J). While there was a negative correlation between *CXCR2* and resting NK cells (*R* = −0.78; Figure 6J). This suggests that key genes may be engaged in APS-associated recurrent miscarriage by influencing the immune microenvironment.

### 3.7 Candidate drugs prediction

**Figure 7:**
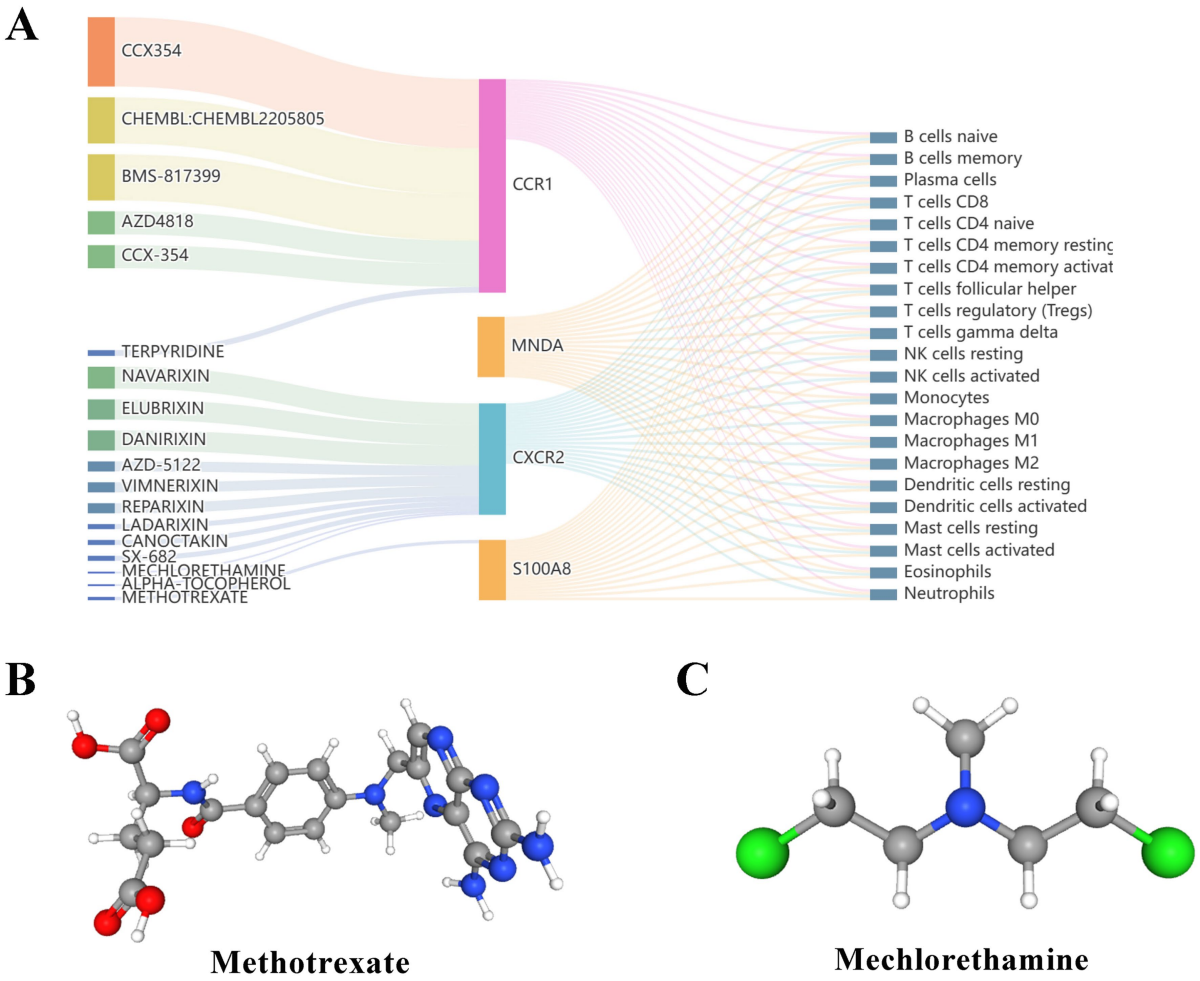
Prediction of candidate drugs. **(A)** Sankey diagram showed the relationship between candidate drugs, genes and immune cells. **(B,C)** The 3D structural tomography of the two approved candidate drugs.

Small molecule drugs with potential therapeutic effects on RM with APS were retrieved from the DGIdb database. Compared with *S100A8* and *MNDA*, *CCR1* and *CXCR2* have a relative abundance of targeted agents and are important potential therapeutic targets for APS and RM and are closely related to immune cells (Figure 7A). Of the top 20 drugs screened, only two drugs are currently approved, and the 3D structures of these two drugs were found in PubChem (Figures 7B,C). Information on the small molecule drug candidates is detailed in Table 4.

**Table 4:** The top 20 drugs screened from the DGIdb database.

Gene	Drug	Regulatory approval	Interaction score
CCR1	CCX354	Not Approved	16.85144397
CCR1	CHEMBL: CHEMBL2205805	Not Approved	16.85144397
CCR1	BMS-817399	Not Approved	16.85144397
CCR1	CCX354	Not Approved	8.425721985
CCR1	AZD4818	Not Approved	8.425721985
CCR1	CCX-354	Not Approved	8.425721985
CXCR2	ELUBRIXIN	Not Approved	7.372506737
CXCR2	DANIRIXIN	Not Approved	7.372506737
CXCR2	NAVARIXIN	Not Approved	5.529380053
CXCR2	AZD-5122	Not Approved	3.686253369
CXCR2	VIMNERIXIN	Not Approved	3.686253369
CXCR2	REPARIXIN	Not Approved	3.686253369
CXCR2	NAVARIXIN	Not Approved	2.457502246
CCR1	TERPYRIDINE	Not Approved	2.106430496
CXCR2	LADARIXIN	Not Approved	1.843126684
CXCR2	CANOCTAKIN	Not Approved	1.843126684
CXCR2	SX-682	Not Approved	1.843126684
S100A8	METHOTREXATE	Approved	1.203674569
CXCR2	MECHLORETHAMINE	Approved	0.670227885
CXCR2	ALPHA-TOCOPHEROL	Not Approved	0.670227885

### 3.8 Validation of key genes

**Figure 8:**
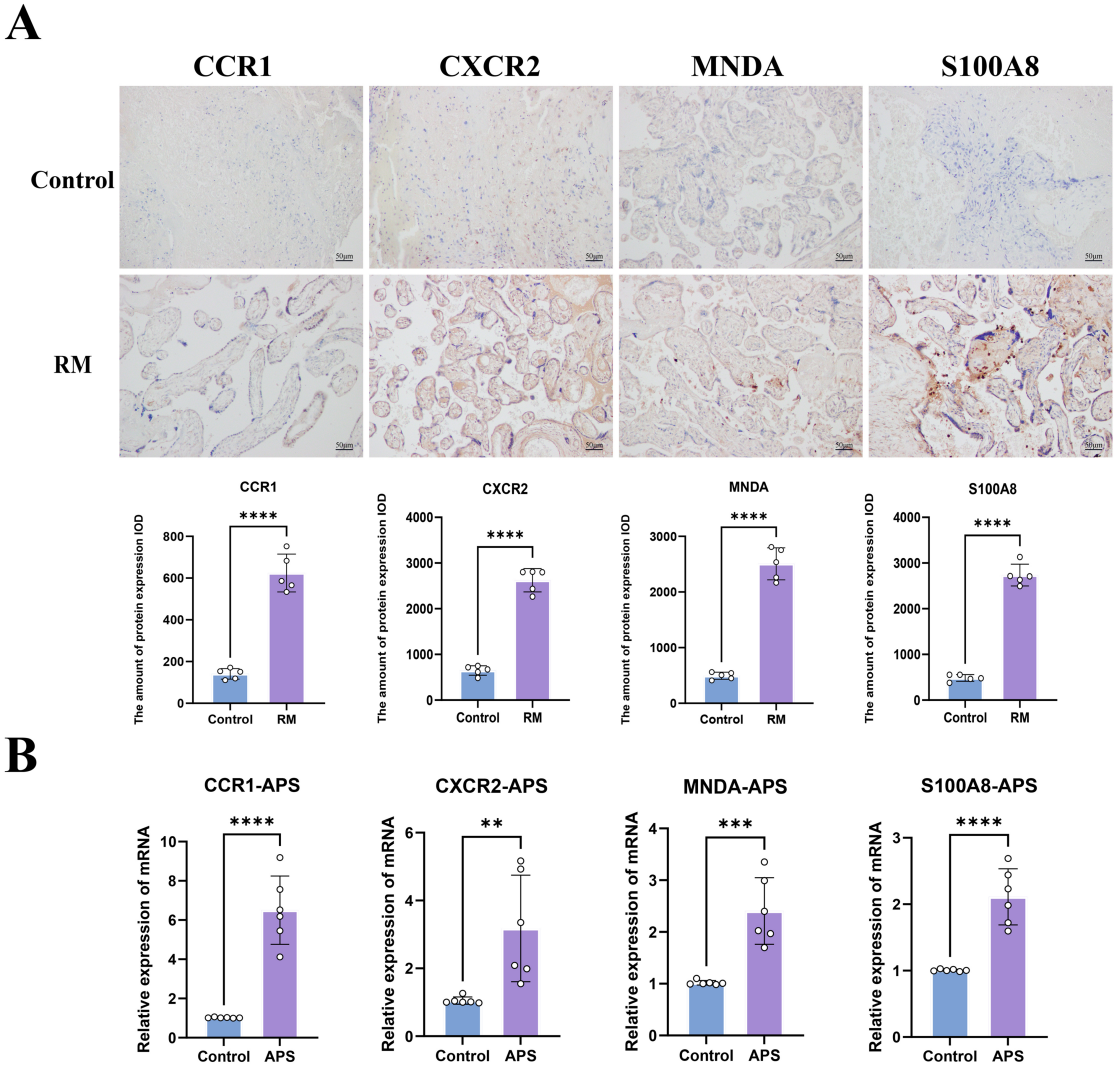
IHC staining and RT-qPCR validated key genes. **(A)** IHC staining of key genes in RM and control groups. **(B)** The RT-qPCR results of key genes in APS and control groups. Noted: ** represents *p* < 0.01, *** represents *p* < 0.001, **** represents *p* < 0.0001.

IHC staining revealed that the protein expression levels of all the key genes were significantly higher in RM than in control group (Figures 8A,B). Similarly, mRNA levels of key genes in the peripheral blood of APS were significantly higher than those of controls (Figure 8C). These results are consistent with our bioinformatics analysis.

## 4 Discussion

As two important diseases affecting women’s reproductive health, the relationship between APS and RM is intricate. There is growing evidence that the two diseases share several common risk factors and that APS may be related to the pathogenesis of RM. (31) Recently, APS has emerged as an important trend in the pathogenesis of RM. (32) Immune cells are vital for embryo implantation, immune tolerance, and embryo growth throughout pregnancy. Disturbances in the immune system of APS patients can lead to cytokine imbalances, which can induce maternal rejection of the fetus, ultimately resulting in a pathologic pregnancy. According to a retrospective study (33), increased subpopulations of Th1 and Th17 cells and decreased levels of Th2 and Treg cells were detected in the blood of APS patients compared to healthy controls. Meanwhile, APS patients had significantly higher levels of several cytokines, including interleukin 1 (IL-1), interleukin 2 (IL-2), interleukin 17 (IL-17), interferon-*γ* (IFN-γ) and tumour necrosis factor-*α* (TNF-α), compared with healthy control population (*p* < 0.05). Furthermore, aCL and aβ2-GP1 were positively correlated with Th17/Treg values in primary APS. Th17 cells produce IL-17, a proinflammatory cytokine that promotes inflammation and maternal-fetal rejection and interacts with Th1 cells, further contributing to the immunopathology of RM. (34) Conversely, Treg cells mediate maternal-fetal tolerance (35), the ability of the embryo to survive in utero without rejection (36). Various cytokines contribute to the maintenance of pregnancy. IL-1, INF-γ, and TNF-α are the major proinflammatory cytokines secreted by Th1 cells. During the window of implantation, pro-inflammatory cytokines facilitate trophoblast invasion and endometrial neovascularization. However, prolonged or excessive exposure to pro-inflammatory cytokines may affect pregnancy and cause miscarriage (37). Wang et al. also suggested that IL-2 and TNF-α in the peripheral circulation of RM with APS may be involved in trophoblast apoptosis and NK cell activation, promoting the development of APS and RM. (38) Therefore, the imbalance in the maternal-fetal immune microenvironment may be significant in APS-induced RM. These findings help to elucidate the molecular mechanisms of APS and RM. However, few researchers have explored the common pathogenesis of APS and RM at the genetic level. By combining multiple public databases, our study identified key genes that can serve as biomarkers or potential therapeutic targets for APS and RM, providing a basis for determining the common mechanisms of APS and RM as well as possible clinical treatments.

In our study, we focused on four key genes to elucidate the relationship between APS and RM. *CCR1* (C-C chemokine receptor type 1), also known as macrophage inflammatory protein-1α receptor, is an important member of the chemokine-C receptor family and is widely expressed in a variety of cells, including tumor cells, myeloid-derived suppressor cells, and monocytes/macrophages (39–41). CCR1 has many ligands and is involved in cell migration, cell differentiation, immune response, immunoregulation and other pathophysiological processes through binding to ligands, such as CCL5 (42–44). Previous studies have revealed that CCR1 mediates the migration and recruitment of monocytes, and transmits the accumulation of macrophages (45, 46). Macrophages are the second most abundant immune cells at the maternal-fetal interface, and decidual macrophages are essential for the maintenance of pregnancy because they are involved in a variety of processes, including immune tolerance, clearance of apoptotic cells, and regulation of trophoblast activity (47–49). Macrophages are categorized into M1 pro-inflammatory and M2 anti-inflammatory types. An increased proportion of M1 molting macrophages is thought to be associated with RM. (50) In drug-induced liver injury, CCL5 directly activates M1 polarization and inhibits M2 polarization through the CCR1-mediated MARK and NF-κB pathways (51). In this study, CCR1 expression was found to be elevated in APS and RM. Hence, the promotion of M1 macrophage polarization may be the mechanism by which CCR1 is involved in the development of RM.

S100A8 (S100 calcium-binding protein A8) and S100A9 (S100 calcium-binding protein A9) assemble into calprotectin, an inflammatory marker that has been found to drive procoagulant platelet formation via platelet glycoprotein Ib alpha chain precursor (GPIbα) (52). It may lead to abnormal thrombus formation under pathological conditions. In addition, calprotectin is involved in a variety of autoimmune diseases, including inflammatory bowel disease and rheumatoid arthritis (53, 54). Recent research revealed that calprotectin induces caspase-1-dependent platelet inactivation by binding to toll-like receptor 4 (TLR4) on the platelet surface and activating the NLRP3-inflammasome, leading to APS thrombocytopenia (55). Several studies have shown a strong association between S100A8 and the development of RM. RNA sequencing revealed upregulation of the S100A8 gene is associated with adverse pregnancy outcomes (56) and elevated S100A8 concentrations were significantly associated with shorter gestation times (57). It has been found that S100A9 levels are elevated in preeclamptic platelets and verified that platelets treated with S100A8/S100A9 show a mild increase in procoagulant activity (58). It is hypothesized that S100A8/S100A9 is an antithrombotic target in preeclampsia. Furthermore, S100A8/S100A9 expression levels were found to be upregulated in placental samples from RM patients (21). According to our study, aberrant expression of S100A8 may be closely associated with autoimmune diseases and pregnancy complications.

*CXCR2* (C-X-C motif chemokine receptor 2) is a receptor for multiple ligands, including interleukin 8 (IL-8), C-X-C motif chemokine ligand 1 (CXCL1), C-X-C motif chemokine ligand 2 (CXCL2) and C-X-C motif chemokine ligand 5 (CXCL5). Although the role of CXCR2 in the pathogenesis of APS and RM has not been investigated, it has been implicated in the development of other autoimmune diseases (59–61). Previous study has revealed that human chorionic villi express CXCR2 (62). IL-8 was also detected to be expressed in human meconium and trophoblast and to promote trophoblastic migration and invasion (63). Meanwhile, there is a genetic association between the rs1126579 polymorphism in the CXCR2 gene and an increased risk of preeclampsia (64). In addition, the CXCR2/CXCL1 axis promotes the recruitment of myeloid-derived suppressor cells (MDSCs) to the decidual tissue and maintains pregnancy tolerance (65, 66). Ma et al. showed a significant attenuation of proliferation and migration of endothelial cells of the decidualized vasculature after treatment with a CXCL1-neutralizing antibody or a CXCR2 inhibitor in experiments with mice (67). The upregulated expression level of IL-8 in primary APS patients with venous thrombosis (68), all of which further support the effect of APS on RM.

MNDA (Myeloid nuclear differentiation antigen) is a member of the interferon-regulated 200 protein family. It has been demonstrated to promote programmed cell death under a variety of experimental conditions. Meanwhile, the researchers found that the expression level of MNDA is critical for the response of embryonic stem cells against DNA damage (69, 70). Currently, there are fewer studies on MNDA in APS and RM. Therefore, the relationships between MNDA, APS, and RM deserve further exploration.

The results of KEGG revealed that CGs were most significantly enriched in neutrophil extracellular trap formation (NETs). Our analysis specifically implicated key NETosis-related genes, such as *CXCR2*. Neutrophil activation and release of NETs are part of the mechanism of interaction between inflammation and coagulation (71). The aβ2-GP1 was shown to activate neutrophils many years ago (72). Various NETs regulatory proteins were associated with APS thrombosis, such as peptidyl arginine deiminase, neutrophil elastase, and myeloperoxidase (73). Neutrophil-driven inflammation promotes disruption of the placental tissue barrier and is associated with fetal cardiac development (74). In the present study, neutrophils are the main infiltrating immune cells of APS. The upregulation of *CXCR2* aligns with enhanced neutrophil recruitment and NETotic activity. These findings further support the possibility that neutrophil activation and NETs may be a common pathogenesis of APS and RM.

Enrichment analysis of key genes suggested that immune disorders are the main mechanism for the development of APS and RM. Immune cell changes are directly related to immune homeostasis at the maternal-fetal interface. Abnormalities in the number and function of immune cells appear to be significantly associated with APS and RM. Our study suggested that key genes may influence the number of immune cells. Eosinophil and Treg cell levels in placental tissues in RM were higher than normal and positively correlated with key genes. Eosinophils mediate parasitic and allergic responses and are often elevated when hookworm-infected women become pregnant (75). However, eosinophil proportions were previously thought to remain constant or decrease during pregnancy (76). Currently there is a lack of research on the relationship between eosinophils and RM. Treg cells suppress immunity and play an important role in embryo attachment and maintenance of tolerance at the maternal-fetal interface (35). It also regulates the phenotype of other immune cells by secreting cytokines (77). Inadequate Treg cells numbers or functional capacity is associated with RM. (78) However, the number of Treg cells was elevated in placenta samples with RM in this study, and follow-up work is needed to further explore the relationship between Treg cells and RM.

Finally, the candidate prediction drugs screened from the DGIdb database were methotrexate and mechlorethamine. Methotrexate is a folate antagonist that has been used to treat a variety of autoimmune diseases (79, 80). It also has teratogenic properties by interfering with DNA synthesis (81). Therefore, whether methotrexate can be used in the treatment of RM is still full of controversy. Mechlorethamine and its derivatives are used as chemotherapeutic agents, mainly in the treatment of leukemia, lymphoma and other malignancies. It can inhibit the growth of cancer cells by cross-linking DNA strands and interfering with division and proliferation (82). However, there is still a gap in the research on nitrogen mustard, APS, and RM. Although the DGIdb database is an effective strategy for finding therapeutic drugs, more evidence from a large number of animal experiments and clinical trials is still needed because these drugs and target genes are only obtained by pure computer prediction.

Nevertheless, because APS is a systemic disease, receiving the limitation of experimental conditions, only the sample database of peripheral blood can be retrieved at present, which is inconsistent with the tissue source of RM, and may be an important factor affecting the results. Second, although the integration of multiple microarray datasets enhances the statistical power and generalizability of our findings, it may also introduce certain limitations. The preprocessing steps, including normalization and conversion to Entrez gene IDs, are necessary to harmonize data from different platforms; however, this process might obscure platform-specific biases or unique biological signals inherent in individual datasets. While our approach prioritized the identification of consistent biomarkers across diverse cohorts, we acknowledge that some subtle yet biologically meaningful patterns may have been attenuated. Future studies could benefit from platform-aware meta-analytical methods or single-cell sequencing technologies to preserve dataset-specific characteristics while still leveraging multi-cohort integration. Furthermore, our validation in RM was confined to placental tissue. While this is the most relevant site for studying the local pathophysiology, it remains unknown whether the expression levels of *CCR1*, *MNDA*, *S100A8*, and *CXCR2* are similarly dysregulated in the peripheral blood of RM patients. Future studies are warranted to investigate their potential as circulating biomarkers for a less invasive diagnostic approach. Finally, the safety and efficacy of the candidate drugs also need to be verified.

In conclusion, through integrated bioinformatics and machine learning, we identified *CCR1*, *MNDA*, *S100A8*, and *CXCR2* as shared key genes between APS and RM. These genes are significantly upregulated and are implicated in dysregulated immune responses, particularly neutrophil activation and thrombo-inflammation, suggesting a common pathogenic pathway. Our analysis highlights the potential of these genes as novel diagnostic biomarkers and therapeutic targets. The inferred involvement of neutrophil extracellular traps (NETs) provides a fresh perspective on the mechanisms linking APS to pregnancy loss. While further functional studies and clinical validation are essential, this work lays a foundational framework for understanding the intricate interplay between autoimmunity and reproductive failure and paves the way for developing targeted strategies to improve pregnancy outcomes in affected women.

## 5 Conclusion

In summary, we identified *CCR1, CXCR2, MNDA* and *S100A8* as shared key genes for APS and RM. They may be good biomarkers for APS and RM. This may provide new insights for the diagnosis and treatment of APS-induced RM in the future.

## Data Availability

The original contributions presented in the study are included in the article/Supplementary material, further inquiries can be directed to the corresponding author.

## Footnotes

^1^ https://www.ncbi.nlm.nih.gov/geo/

^2^ https://www.metascape.org

^3^ https://string-db.org

^4^ https://ctdbase.org

^5^ https://cibersortx.stanford.edu/

^6^ https://www.dgidb.org

^7^ https://pubchem.ncbi.nlm.nih.gov/
