# Supplementary figures and images for: Identification of shared biomarkers and potential therapeutic targets for antiphospholipid syndrome and recurrent miscarriage by integrated bioinformatics analysis and machine learning
Source: Front Med (Lausanne). 2025 Sep 23;12:1639277. doi: 10.3389/fmed.2025.1639277 (PMC12500650; doi:10.3389/fmed.2025.1639277)

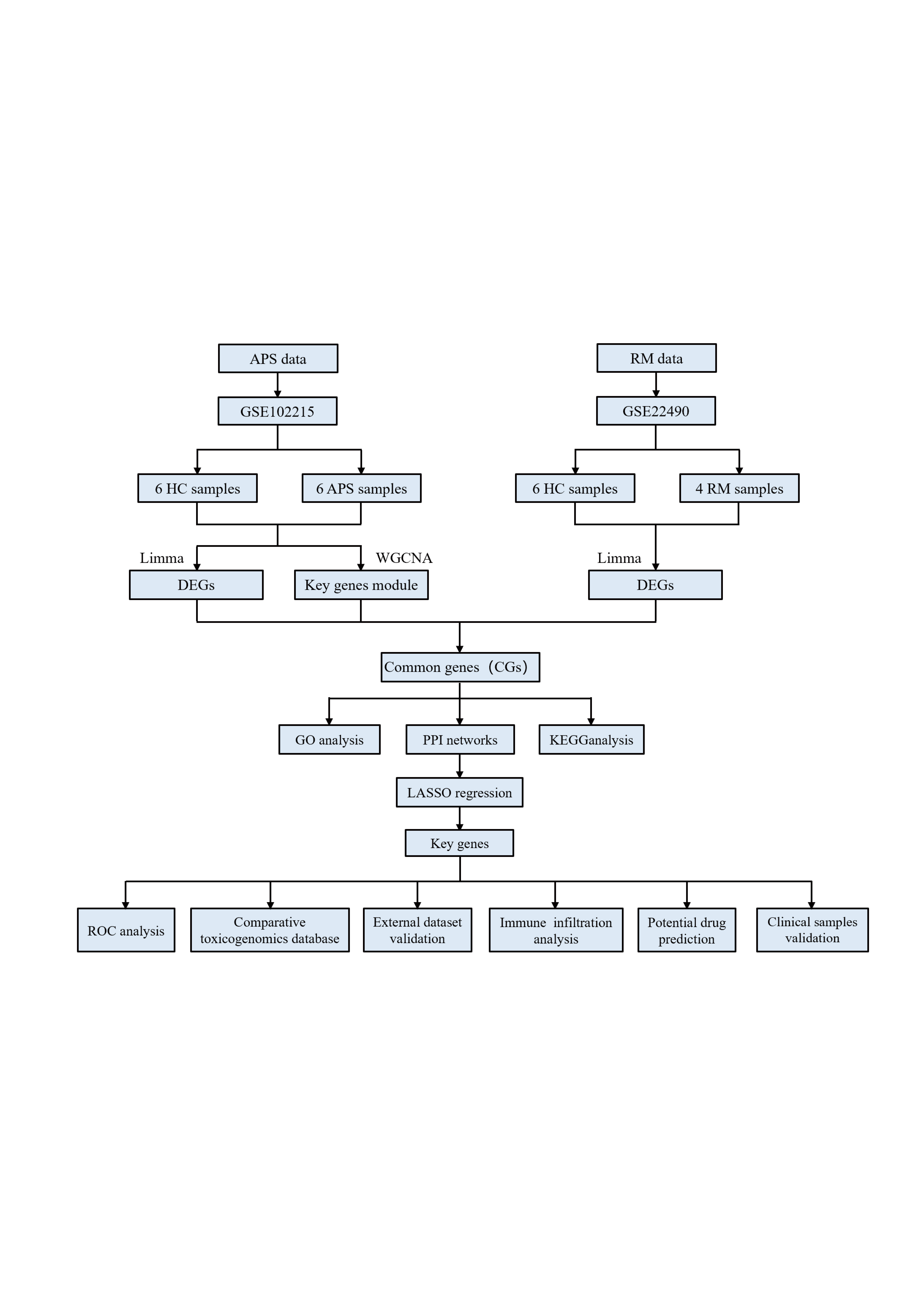

Supplement: Supplementary file 1 [file Image_1.jpeg]
